# ANAD: Arabic news article dataset

**DOI:** 10.1016/j.dib.2023.109460

**Published:** 2023-07-29

**Authors:** Mohammed Altamimi, Abdulaziz M. Alayba

**Affiliations:** Department of Information and Computer Science, College of Computer Science and Engineering, University of Ha'il, Ha'il, 81481, Saudi Arabia

**Keywords:** Arabic news articles, Data analysis, Classification, Natural language processing (NLP)

## Abstract

In this paper, we present a modern standard Arabic dataset based on Arabic news articles collected over a one-year period from 01/01/2021 to 12/31/2021. In total, from 12 Arabic news websites, over 500,000 articles were collected, the selection of which was driven by a variety of topics, including sports, economies, local news, politics, tech, tourism, entertainment, cars, health, and art. The development of this dataset will enable data scientists to explore and experiment effectively in the field of natural language processing, and the dataset can also be used to develop machine learning and deep learning models to classify articles according to topic. The dataset is available for download at

https://github.com/alaybaa/ArabicArticlesDataset/tree/main.

**Specifications Table**

**Table:** 

Subject	Data science
Specific subject area	Machine learning, Deep learning, Natural language processing (NLP), Text classifications, Text summarization, Arabic dataset.
Type of data	Text files
How was the data acquired	The dataset was acquired from 12 Arabic news websites using web-scraping tools. Python is the main language used to collect the articles, with two main packages used: Requests [1] and BeautifulSoup [2].
Data format	Annotated text dataset (.txt)
Description of data collection	The Arabic articles were retrieved over a period of one year, between 01/01/2021 and 12/31/2021, using web-scraping tools. We collected the articles from different news websites focusing on different categories or topics, as well as articles on such topics as sport, economies, local news, politics, technology, tourism, entertainment, cars, health, and art. Each article is annotated in the dataset according to the website categories of each, and several pre-processing steps were performed to ensure the datasets were cleaned.
Data source location	The article sources are: alwatan.com.sa, akhbaar24.argaam.com, ienalwatan.com, neprass.org, aleqt.com, at-magazine.com, aitnews.com, aawsat.com, alarabiya.net, bbc.com/arabic, ksa.motory.com/ar, and almuraba.net.
Data accessibility	The data are available freely for download using the direct URL below https://github.com/alaybaa/ArabicArticlesDataset/tree/main. https://zenodo.org/badge/latestdoi/597211607. Data identification number: 10.5281/zenodo.8173006.

## Value of the Data

• The dataset contains 500,725 articles annotated according to their topics.
• The articles are written in modern standard Arabic style and represent 10 distinct categories.
• The dataset is filtered of any extra details, apart from the article title and body.
• All duplicate articles have been removed.
• All irrelevant details in each article have been removed, such as the Author Name(s), Keywords, article date, etc.
• The articles are between 275 and 107,572 characters in length.

## 1 Data Description

The dataset has 10 categories, including sport, economies, local news, politics, technology, tourism, entertainment, cars, health, and art, and the selected websites represent four major news categories: a TV channel website, a newspaper website, electronic media website, and a journal website. Articles are collected according to the categories available on each of the websites, and each article is annotated according to the category in its news portal source. The dataset is then uploaded to GitHub. The articles are organized according to the website source; they are classified according to the category to which they belong. The dataset is suitable for research and is useful for building machines and deep learning models. Further, it can be used to perform different NLP tasks, such as text classification, text generation, sentence similarity, and text summarization. The distribution of articles per category for each website is summarized in Table 1.

**Table 1:** Distribution of articles per category.

Website	Categories	Number of Articles
alarabiya.net[3]	sport, economies, politics, technology, tourism, health, and art	134,471
bbc.com/arabic[4]	politics, health	1,400
aawsat.com[5]	sport, technology, tourism, cars, health, art	9,084
aleqt.com[6]	sport, economies, technology, tourism, art	99,314
alwatan.com.sa[7]	sport, economies, local news, politics	33,357
akhbaar24.argaam.com[8]	sport, technology, tourism, entertainment, cars, health	127,123
ienalwatan.com[9]	sport, economies, local news, health, art	38,414
neprass.org[10]	technology, tourism, health	204
Alsyahaalarabia.com[11]	tourism	5,937
aitnews.com[12]	technology	38,741
sa.motory.com/ar[13]	cars	1,334
almuraba.net[14]	cars	11,337

Of the data representing the TV channel website, we selected *AlArabiya* [3] website, an international Arabic news channel. It was founded in 2003 and operated from Dubai, but the operation was recently moved to the capital city of Riyadh, Saudi Arabia. It has a large audience in the Middle East, and it focuses on diverse news categories, such as politics, economies, and sports. From this website, 134,471 articles in total are collected, and they are annotated according to the internal pages on the website. The articles are classified into seven categories, as follows: sports, economies, politics, technology, tourism, health, and art.

In addition, the Arabic version of the *BBC* [4] is another example of a TV channel website that we considered in our dataset. The channels were launched in 1990 and based in London, whereas the website is derived from the mother *BBC*, which is manly spoken in English. The website is more popular, with a variety of news articles; however, according to our selection criteria, only 1,400 articles were collected from this website representing two categories: politics and health.

For data that represent a newspaper website, we utilized the *Asharq Alawset* newspaper [5]. The Arabic international newspaper, founded in London 1978, generally had an influence on the Middle East region. In this dataset, we collected 9,084 articles mainly classified as related to sport, technology, tourism, cars, health, and art categories.

Furthermore, the *Al Eqtisadiah* [6] newspaper is another source considered in our dataset. Ranked the second-most popular newspaper in Saudi Arabia and the eighth-most popular in the Middle East according to Forbes Middle East [15], it was founded in 1992 in the capital city of Saudi Arabia, and it focuses on economic and marketing news at the local, regional, and international levels. The dataset in this category consists of 99,314 articles, which are tagged in up to five categories, including sport, economies, technology, tourism, and art.

Another source from which we collected articles is *Alwatan Newspaper* [7]. Founded in 2000 in Abha, a city located in the south of Saudi Arabia, *Alwatan Newspaper* offers local news with a focus on the southern region of Saudi Arabia [16]. The newspaper website was launched in 2010, from which we have collected 33,357 articles related to sport, economies, local news, and political news.

Electronic media has recently gained more popularity due to the ease of access, availability, and increased diversity in recent years. Electronic media uses digital contents, such as video recordings, audio recordings, and multimedia presentation, as opposed to newspaper media, which uses printed materials. One example of electronic media is *Akhbaar24* [8], as it focuses on local Saudi Arabian news and events; as such, 127,123 articles were gathered from this website. The articles are tagged with six categories, such are sport, technology, tourism, entertainment, cars, and health. Furthermore, another electronic news website, *Ien Alwatan* [9], also focuses on local Saudi Arabian news. From this website, we collected 38,414 articles related to sports, economies, local news, health, and art. In addition. *Neprass* [10] is another electronic news website utilized in our dataset, as it focuses on technology, tourism, and health articles. In total, 204 articles were collected from this website.

Finally, we also considered single-topic websites, which focus usually on one news issue, in our dataset to increase the quantity of articles in some categories. For instance, the *Arabic Tourism Journal* [11] website focuses on articles related to tourism news. Thus, 5,937 articles in the tourism category were collected, in total. In addition, the *Arabic Portal for Technical News* [12] is a website specializing in technology news and we collected 38,741 technology articles. Lastly, *Motory* [13] and *Almuraba* [14] are both websites that highlight the latest trends in motors and cars; from both websites, we collected over 12,000 articles related to the latest news about cars.

**Fig. 1:**
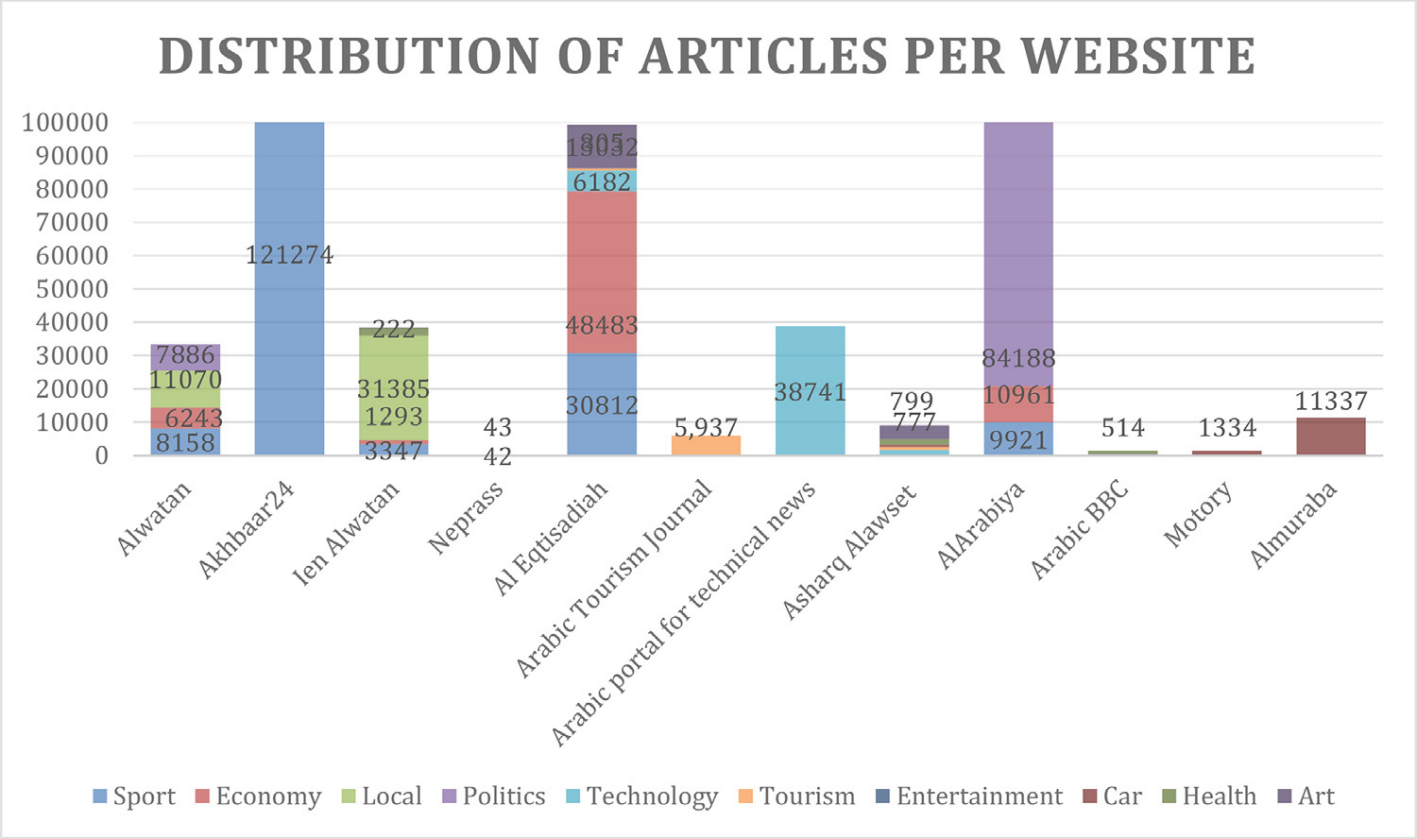
Distribution of articles per website.

**Fig. 2:**
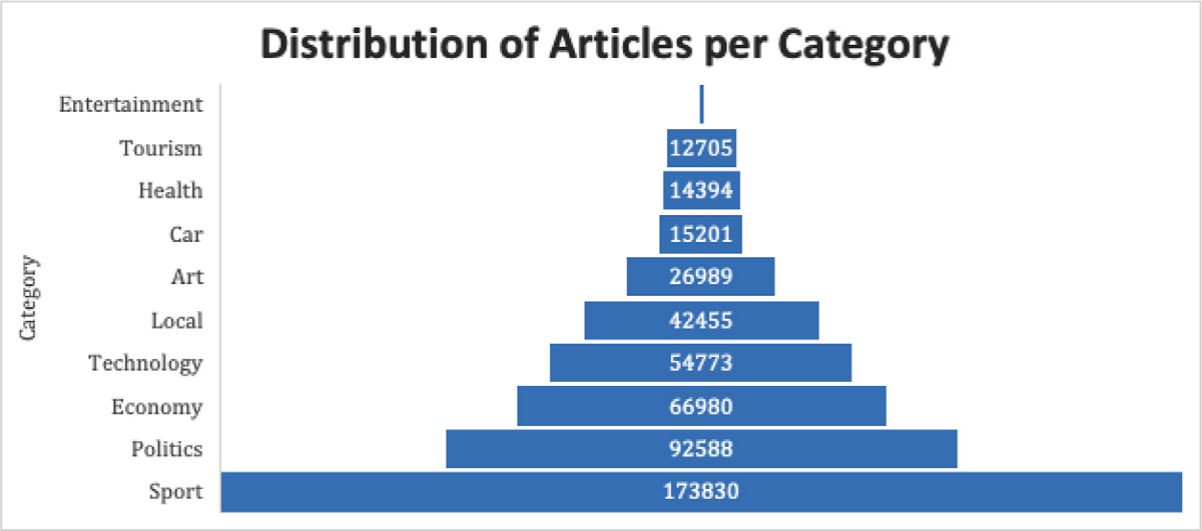
Distribution of articles per category.

Figs. 1 and 2 show the distribution of articles according to the category of each website. This clearly shows that the distribution of these articles reflects five categories: sports, politics, economies, tech, and local news.

## 2 Experimental Design, Materials and Methods

The dataset was retrieved from websites during the period between 01/01/2021 and 12/31/2021 using web-scraping tools, as well as Python, which has many packages, including Requests and BeautifulSoup, which support the retrieval of data from the web. Requests is an integral python module that deals with HTTP pages. It involves many methods, including get, post, delete, head, etc., the former of which was used to retrieve HTML pages of the mentioned sources. In addition, BeautifulSoup, another package used, can extract information from HTML pages, but the only information required from this dataset is the article title and article body. In the collection process, several criteria were considered to retrieve articles from newspaper websites, as follows:

- The structure of the news website is based on pages that loop through other pages.
- The newspaper addresses one of the targeted categories, i.e., sport, politics, etc.
- The news website is retrievable, as some websites are strictly unretrievable.

Algorithm 1 represents the general pseudocode that has been used to collect the articles from the websites’ sources.

**Algorithm 1:** Article collection process.

1 Create an Output Folder 2 Count = 1 3 For Article Pages in (First page, Last Page) do 4 Get Current Page URL 5 Parse HTML of Current Page 6 Find all article links in the Current Page 7 For Article Tags List in (Current Page) do 8 Find Current Article URL 9 Get Current Article URL 10 Parse HTML of the Current Article 11 Find Title of the Current Article 12 Find Body of the Current Article 13 Remove any characters that irrelevant to the article 14 Open txt File (“Name of Category” + Count) 15 Write Title \n \n Body into a txt file 16 Close txt File 17 Count +=1 18 End For 19 End For

**Line 1 of**Algorithm 1 involves creating folders for the retrieved articles, and each folder was named according to the category, e.g., economies, technology, art, etc. **Line 2** is a counter variable, and it begins from one. **Line 3** is a for loop that starts from the first page and continues to the last page of a single category, whereas **line 4** involves getting the HTML code of the current page using the Requests library [1]. **Line 5** involves converting the HTML page to be passable and traceable using the BeautifulSoup package [2], and **line 6** contains the *find all* function of the BeautifulSoup package [2]. It searches for certain HTML tags with specific attributes as well. In this line, we list all the article tags of the current page. **Line 7** is a for loop in the list of retrieved article tags, while **Line 8** finds the current article URL tag. **Lines 9 and 10** are similar to **lines 4 and 5**, as they focus on the article pages. Meanwhile, **lines 11 and 12** find both the title of the article and the body of the article, respectively, and **line 13** eliminates any irrelevant characters or HTML tags that appear in either the title or the body. At the beginning of each category retrieval, we collected a random number of articles. Then, we checked the output for any text or characters unrelated to the article. **Line 14** opens and creates a new text file in .txt format, the name of which contains the category name and counter number. In addition, we used a UTF-8 character-encoding system for the Arabic text. **Line 15** writes the retrieval results, which include the title of the article and the body, and we used two new lines after the title and before the body. **Lines 16 and 17** close the .txt file and increase the counter by one,respectively. Finally, **lines 18 and 19** are the end of the for loops, that is, the for loop in **line 7** and the for loop in **line 3**, respectively.

## 3 Filtering the Dataset and Challenges

During the retrieval process, we considered some unwanted information, such as image tags, other HTML tags and characters, and other quick links at the ends of articles. Thus, to prepare and improve the quality of the dataset, the following processing steps were applied to all articles:

- A random check of all different categories and newspapers to identify any unwanted information. For example, quick links in certain articles have been identified due to different HTML patterns over time. In another instance, the author's name, and the location are placed at the beginning of the article's body. Both were removed to avoid repetition, which can affect classification tasks.
- Any article sized less than 500 bytes was deleted, because the length is too short.
- There is duplication of some articles due to the long period of data collection, as well as the interruption when running the code, even though there was exceptional handling of the code. Any duplicate articles within the same category and from the same newspaper were removed.

In the retrieval and filtering process, there were many challenges, as follows:

- Observing the HTML tags and identifying the required tags for different newspaper websites such as, the title, the body of the article and the hyper link that navigate to the next pages.
- Identifying newspaper web pages that are structured based on pages.
- Many websites block requests that come from a programming script without a valid web browser.
- There are many unretrievable resources, and on other web sites, only the first page is retrievable.
- The structures of some web sites are changed, where the bodies or titles of the old articles contain unwanted information, e.g., newspaper name, author name(s), etc.
- Some categories contain a significantly large number of articles, and identifying duplicates is highly challenging.

## 4 Sample of Articles

**Figure:**
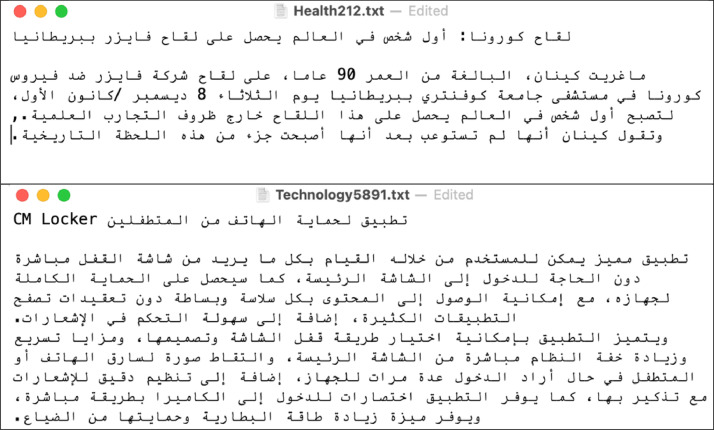

## 5 Limitations

Not applicable.

## Ethics Statements

The authors have read and followed the ethical requirements for publication in Data in Brief and confirm that the current work does not involve human subjects, animal experiments, or any data collected from social media platforms.

The authors did not need permission to use the language data from the respective news sites.

## CRediT authorship contribution statement

**Mohammed Altamimi:** Conceptualization, Methodology, Software, Data curation. **Abdulaziz M. Alayba:** Writing – original draft, Visualization, Investigation, Supervision, Validation, Writing – review & editing.

## Data Availability

ANAD: Arabic News Article Dataset (Original data) (Github).
